# Making species checklists understandable to machines – a shift from relational databases to ontologies

**DOI:** 10.1186/2041-1480-5-40

**Published:** 2014-09-08

**Authors:** Nina Laurenne, Jouni Tuominen, Hannu Saarenmaa, Eero Hyvönen

**Affiliations:** Semantic Computing Research Group (SeCo), Department of Media Technology, Aalto University, P.O. Box 15500, 00076 Aalto, Espoo, Finland; Digitarium, University of Eastern Finland, P.O. Box 111, 80101 Joensuu, Finland

**Keywords:** Scientific name, Taxonomic concept, LSID, HTTP URI, Ontology, Semantic web, Linked data, Species checklist

## Abstract

**Background:**

The scientific names of plants and animals play a major role in Life Sciences as information is indexed, integrated, and searched using scientific names. The main problem with names is their ambiguous nature, because more than one name may point to the same taxon and multiple taxa may share the same name. In addition, scientific names change over time, which makes them open to various interpretations. Applying machine-understandable semantics to these names enables efficient processing of biological content in information systems. The first step is to use unique persistent identifiers instead of name strings when referring to taxa. The most commonly used identifiers are Life Science Identifiers (LSID), which are traditionally used in relational databases, and more recently HTTP URIs, which are applied on the Semantic Web by Linked Data applications.

**Results:**

We introduce two models for expressing taxonomic information in the form of species checklists. First, we show how species checklists are presented in a relational database system using LSIDs. Then, in order to gain a more detailed representation of taxonomic information, we introduce meta-ontology TaxMeOn to model the same content as Semantic Web ontologies where taxa are identified using HTTP URIs. We also explore how changes in scientific names can be managed over time.

**Conclusions:**

The use of HTTP URIs is preferable for presenting the taxonomic information of species checklists. An HTTP URI identifies a taxon and operates as a web address from which additional information about the taxon can be located, unlike LSID. This enables the integration of biological data from different sources on the web using Linked Data principles and prevents the formation of information silos. The Linked Data approach allows a user to assemble information and evaluate the complexity of taxonomical data based on conflicting views of taxonomic classifications. Using HTTP URIs and Semantic Web technologies also facilitate the representation of the semantics of biological data, and in this way, the creation of more “intelligent” biological applications and services.

**Electronic supplementary material:**

The online version of this article (doi:10.1186/2041-1480-5-40) contains supplementary material, which is available to authorized users.

## Background

Research on biodiversity requires integrating data from distributed heterogeneous sources, such as scientific literature, observations, and biomedical resources. Data is often presented using a variety of terms, vocabularies, and languages, which presents a barrier to interoperability and makes data reuse and integration a challenge for both human users and machines.

Scientific names are important for interlinking information about taxa in all fields of the Life Sciences. A taxon is a group of one or more organisms whose members are considered evolutionarily related to one another; a taxon typically has a name and rank, i.e., a species, genus, etc. Taxon names are especially necessary when indexing biological information and cataloguing biodiversity. The nature of names, whether important or problematic, has recently been re-examined by several researchers [1–6]. Difficulties arise when a particular taxon can be referred to using multiple names, since scientists’ opinions differ on how evolutionary units should be organised into classifications. Also, researchers may use the same name with a different meaning when referring to taxa. Well-conducted taxonomic studies may be 250 years old and still useful but in most cases, the perceived boundaries of taxa have been revised several times after the original publication. Contrary to popular belief, a generally agreed-upon, single taxonomy of organisms does not exist, and this fact is directly reflected in the scientific naming system through the various usages of names. For a taxonomist, a scientific name is a label that mirrors an evolutionary hypothesis that is under continuous testing. There will never be a commonly agreed upon single taxonomy and there will always be multiple competing current taxonomic views. Nevertheless, efforts are made to provide usable taxonomies for non-taxonomists.

Checklists are species catalogues where taxa are organised hierarchically according to an author’s current view of a classification. The coverage of a species checklist varies from a geographically limited area to a worldwide list, and it typically focuses on a particular organismal group. An author’s view of research results is thus inevitably emphasised, which opens the lists to interpretation if they lack sufficient taxonomic details. A regional species list indexes taxa of a given area, but it can also contain additional information. For example, Fauna Europaea [7] and the Atlas of Living Australia [8] provide distribution maps and visualisation tools. The database Encyclopedia of Life (EoL) [9] covers the whole world and has a considerable amount of species information. Also, unlike most resources, it supports multiple classifications since data providers can upload differing taxonomies into the system.

Checklists were previously only published in journals (static lists), but up-to-date checklists (dynamic lists) are increasingly available on the web. For example, the most notable database, Catalogue of Life (CoL) [10], aims to include all known species and currently contains nearly 1,352,112 species from 132 taxonomic datasets (2013 Annual Checklist). The database of zoological names ZooBank [11] currently has 101,777 nomenclatural acts. The Global Biodiversity Information Facility (GBIF) [12] has made an effort to stabilise name usage by setting up a Checklist Bank [13] for storing names and information about them. The widely used Taxonomic Concept Transfer Schema (TCS) [14] specifies the format (XML), in which taxonomic information is presented when exchanging data. Darwin Core (DwC) [15, 16], created by Biodiversity Information Standards (TDWG) [17], is a standardised form of presenting biological information. The metadata elements in DwC are not strictly defined as the format and the element values are not fully specified. This means that the interoperability of DwC records is not achieved if the elements are not used in a consistent way. For example, a taxon name may be a literal value or referred to using a URI. Darwin Core Archive (DwC-A) [18] is a data standard for producing a self-contained dataset for sharing species-related data, such as occurrence records and checklists. The CSV (Comma-Separated Values) data files of an archive are organised in a star-like manner, with one core data file and possible extensions, e.g., for vernacular names or distribution data.

The scope of biomedical resources differs from checklists because the focus is on a gene or a cell level. Nevertheless, the name question remains relevant because scientific names are used for linking information. Currently, the National Center for Biotechnology Information (NCBI) [19] provides a single robust consensus hierarchy of taxa constructed by experts, but NCBI ambitiously seeks to build a topology based on monophyletic groups, i.e., taxa derived from a common ancestor. NCBI allows flexibility in the acceptance of informal names and surrogate names can be used when contributing data and searching for taxa [5]. The majority of the submitted DNA sequences do not have a binominal scientific name because specimens are not identified into a species level at the time of submission or only surrogate names are used [5]. The significance of DNA sequence data is increasing due to the rapid development of molecular methods that are applied in constructing evolutionary hypotheses and barcoding biodiversity. Consequently, descriptions of new species based on molecular evidence result in an increased number of species in checklists.

A major source for ambiguity in scientific names is that they change over time. One of the most common types of change concerns a Linnean binominal name combination. The genus of a binominal name changes when a species is moved to another genus. For example, the parasitic wasp species *moscaryi* once belonged to the genus *Tetraconus*, but as a result of a taxonomic revision that synonymised two genera, its new name combination is *Monomachus moscaryi*[20]. Synonymisation happens due to assessments of the identity of types (i.e., typically a physical specimen to which a scientific name is attached). If two or more taxa are lumped, the older name remains valid but with a changed taxonomic circumscription, and the more recent names become its synonyms. Consequently, there is more than one name pointing to the taxon, and the taxonomic concept associated with the older name changes. The opposite situation is the split of taxa, where one taxon is divided into two or more taxa. The divergence between a name and its meaning is characteristic of taxonomy, because a scientific name does not necessarily change despite the fact that taxon boundaries are redefined. Researchers can also classify the same species in various ways, thus leading to the existence of multiple name combinations.

Berendsohn [21] introduced the concept of a potential taxon, which is a scientific name with information on a circumscription. He proposed the term “secundum” (abbr. “sec”) be attached to a scientific name when referring to a particular taxonomic circumscription. This was a concrete suggestion on how to interlink differing taxonomic views while continuing to retain the adequate taxonomic information in databases [22]. Having information on circumscriptions in databases is an improvement, but machine-readable semantics need to be used in order to enhance the machine-processability of taxonomic information.

In this paper we present two models for describing taxonomic information in a machine-processable way. The first model describes species checklists as a relational database and the second one is further developed representation of taxonomic information using Semantic Web technologies. We explore the reasons for moving away from relational databases towards semantic technology, and we also discuss options for managing scientific names as they change over time.

### Towards semantic handling of biological names

A biologist understands the semantics of scientific names by reading scientific literature, but computers require explicit identifier systems and data models to process semantics. It is obvious that persistent identifiers for taxa should be used instead of ambiguous name strings to increase the processability of scientific names. Using identifiers allows information to be connected unambiguously, which enables interoperability between systems. Furthermore, there is a need to interlink taxa between the different versions of checklists as they are updated. Otherwise, data indexed using an earlier version of a checklist cannot categorically be found using a later version of the checklist.

#### Recognising taxa using identifiers

**Figure 1:**
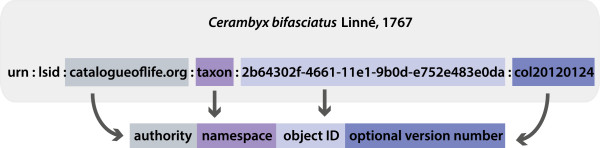
**The structure of an LSID.** An LSID of a cerambycid beetle species obtained from the Catalogue of Life database.

The most commonly used identifiers in biology are Life Science Identifiers (LSID) [23]. An LSID consists of six parts (Figure 1): the first two indicate that the type of URN (Uniform Resource Name) is an LSID, the third part expresses the authority, and the fourth specifies the namespace (which specifies the type of an LSID, e.g., scientific name, living thing, picture, or museum specimen), the fifth points to the object ID, and the optional sixth part is for versioning information. An LSID can be accommodated to a single name or to a set of taxonomic details, depending on its purpose [2, 24]. For example, identifiers are given to scientific names in the World Register of Marine Species [25], but in the Catalogue of Life [10] they are given to taxonomic concepts. The Universal Biological Indexer and Organizer (uBio) [26] has 11,106,374 namebank records where LSIDs are used for referring to taxonomic concepts [6]. Also, an RDF (Resource Description Framework) representation [27] is provided but some of the essential information is expressed as literals (a classification, taxonomic rank and a typing of resources) instead of URIs, which hampers machine-processability.

The data carried by an LSID is obtained using a specific resolver. Locating the resolver via the Domain Name System (DNS) of the Internet requires that the resolver be configured in a DNS SRV record (DNS service record) of the domain used as the authority part of an LSID. LSIDs can also be used without a resolver if they are presented as HTTP URIs using an LSID HTTP proxy. According to the TDWG guidelines for using identifiers, an LSID resolver should return metadata about the requested resource in RDF form [27]. The application of LSIDs in the Catalogue of Life is thoroughly discussed by Jones et al. [2]. GBIF has published recommendations for the adoption of LSIDs and HTTP URIs [28, 29].

The URN scheme applied to LSIDs is a URI scheme standardised by the Internet Assigned Numbers Authority (IANA) [30]. HTTP is also a URI scheme, but there is a fundamental difference between URNs and HTTP URIs. HTTP URIs are based on the DNS, where the global uniqueness of identifiers is guaranteed by the DNS infrastructure, which also facilitates addressing and retrieving information about HTTP URIs. In contrast to URNs, separate web services are not necessary to manage identifier creation or resolve them for data retrieval because these functions are already available in the infrastructure of the web. As a result, HTTP URIs are used as the identifier mechanism for the Semantic Web and Linked Data [31]. In addition, the form of an HTTP URI is flexible because it does not have strictly defined parts like LSIDs. HTTP URIs allow linking data across the web on the basis of the meaning of concepts that are identified with HTTP URIs, which enables the creation of the Web of Data.

LSIDs were the first attempt to solve the name problem, but due to the rapid development of Semantic Web technologies, the trend now favours standardised web technology. The main differences between LSIDs and HTTP URIs are presented in Table 1. The technology applied does not solve the problem of the divergence between a name and its meaning, but it does provide an appropriate solution for publishing and interlinking data in an interoperable way on the web.

**Table 1:** **The main differences between LSIDs and HTTP URIs**

	Life science	HTTP URIs
	identifiers	
Standardised by	Object Management	Internet Engineering
	Group [32]	Task Force [33]
Reuse existing	Defines a new	Uses an
URI schemes	URN subscheme	existing scheme
Data retrieval/	Specific resolving	Uses existing
dereferenceability	service needed	web technology
		(DNS, web servers)
Structure of identifier	Strict	Flexible
Linked Data compatibility	No	Yes

Both LSID- and HTTP URI-based checklists can be published for humans via a user interface and for machines as APIs (Application Programming Interface) to provide access to the data in multiple ways. For example, the user interface can be used to check a valid name for a taxon and browse a classification. The same information can be obtained using a specialised API, but more general query interfaces can also be provided. In Linked Data, an API for reading the RDF description or a human-readable HTML page for a resource is typically provided, as well as a general purpose endpoint service that can be queried using the Semantic Web query language SPARQL. In addition, checklists can be made available as downloadable files [31].

#### Semantic modelling of taxonomies

On the Semantic Web, taxonomies are represented using RDF resources, i.e., entities with URI identifiers, and explicit relations between them. A relatively new approach is to express taxonomic information as an ontology. The first ontology model for a taxonomic classification was presented by Schulz et al. [34], with taxa organised into a single hierarchy. Franz and Peet [35] and Franz and Thau [36] have offered further insight into the issues of taxonomic ontology modelling. So far, a few taxonomic ontologies have been published in the NCBO BioPortal [37–41] and the ONKI ontology service [42]. The most comprehensive of them is the NCBI Organismal classification [41], which contains more than 352,000 taxa in a single hierarchy. Common to the classifications in the NCBO BioPortal is that the hierarchy is constructed using *subclassOf* (isA) relations and presented in the OBO ontology language [43]. TaxonConcept.org [44] tackles the name problem of taxonomic information in practice and shows how to publish the information as Linked Open Data. It also demonstrates how data from external sources are integrated and investigates how to combine taxonomic concepts with specimen data. However, some of the important information about names are described as literals, e.g., the classification of taxa. Also, the taxonomic change types are not described (split or lump of taxa). The Taxonomic Meta-Ontology TaxMeOn [45, 46] aims to respond to the practical needs of managing biological names over time, and it links taxonomic information to names. This meta-ontology differs in that it offers a greater level of detail and supports differing classifications.

An increasing number of ontologies are available and therefore ontology evolution has become an important issue. The world – and our conceptualisation of it – is continually changing, which makes ontology versioning essential [45, 47, 48]. Existing data that refer to a concept should be kept consistent when its meaning changes or when it is removed from an ontology. Data described using different versions of an ontology then can be integrated by utilising mappings (alignments) between the ontology versions [49]. Khattak et al. [50] document ontology evolution by keeping a log of changes in concepts. Small changes in an ontology are grouped into sets, which can later be used to revert to previous stages. An alternative solution is to recognise concept changes instead of versioning an ontology. Wang et al. [51] show how the changes in concepts and their impacts can be identified automatically by comparing the concepts both extensionally and intensionally in cases where they do not have fixed identifiers.

## Methods

In order to develop two models for presenting taxonomic information in a machine-processable way, four design principles were applied to satisfy the following conditions:

1. use as few terms as possible to express as much information as possible in the schema of the model. The taxonomic terminology and its usage is established in biology, and the terms are used in consistent way. As few new terms as possible are introduced.
2. focus on a restricted domain, that is, scientific species checklists including all taxonomic information and excluding any other taxon-related information (e.g., distribution).
3. support information on various levels of granularity, as the source material is heterogeneous in its level of detail.
4. accept all views of taxonomy equally legitimate regardless of the time they were disseminated.

The focus of the models is in representing the taxonomic relations between taxa in a single checklist (classification, synonymy), in different checklists (mapping taxonomic concepts) and in individual versions of a checklist (managing taxonomic changes).

The datasets utilised in the study consist of 20 published species checklists that cover mainly northern European mammals, birds and several groups of insects and assemble ca. 78,000 taxon names (Additional file 1). Two models are applied to the same datasets. Name mappings between the checklists are provided for eight families of papilionoid and hesperioid butterflies.

## Results

### Taxonomic database

**Figure 2:**
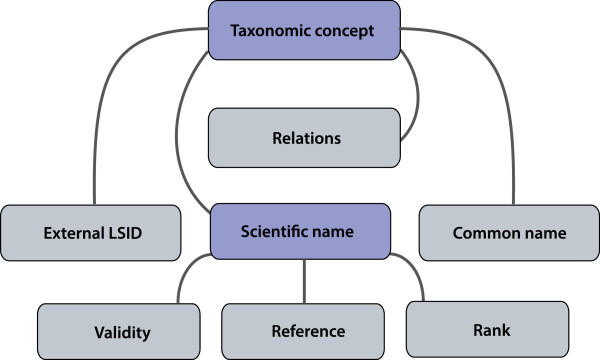
**A simplified structure of the relational taxonomic database.** The boxes illustrate the tables of the database, and the lines present the relations between them. LSIDs are given to taxonomic concepts and scientific names (illustrated with a darker colour). Taxonomic concepts are linked to each other using the relations described in Table 3 and each concept is linked to a scientific name. External LSIDs and common names are connected to the concepts. An author reference, validity, and a taxonomic rank are assigned to the scientific names.

The main elements of the Taxonomic Database (Figure 2) [52] are a binominal scientific name and a taxonomic concept that connects the names that refer to the same taxon. Each concept is identified with a concept LSID. In addition, three other attributes are assigned to the scientific name: 1) a reference to the original publication (author name and year of publication) in which the taxon description was first published, 2) a status of a name indicating its validity in the checklist, and 3) a taxonomic rank expressing level in a hierarchical classification (species, genus, etc.). A taxonomic hierarchy between scientific names is constructed using a hierarchical part-of relation.

An LSID that is obtained from an external source can be assigned to a taxon concept as an attribute. Common names in multiple languages can be connected to the concept, but no taxonomic rank can be specified for them. In order to recognise the orthographic variants of scientific names, LSIDs are accommodated to the names as well.

A new LSID is given to a concept if it changes, such as a taxonomic change, an addition or removal of a synonym, or a change in relations between taxa. An LSID is assigned to a new taxon when added to a dynamic checklist. LSIDs are versioned in the case of minor changes using the optional part of the identifier. The decision whether to create a new object identifier of an LSID or a new version is made by a maintainer.

Taxa can be searched using a complete or partial scientific name via a user interface, and the system returns a currently valid name and its synonyms. If the taxon is found in other checklists, their interrelations are also described. The information is also provided as an RDF representation for machine consumption. Only the latest versions of dynamic checklists can be seen in the system. However, older ones are stored internally in the database.

Taxonomic concepts are linked on the basis of their equivalence at a species level, but at higher levels the alignment of taxa is based on the species content. For instance, two species that have the same name and the same authorship citation are linked as congruent by default, but two genera are linked as congruent only if the species belonging to the genera are the same. The reasons for treating species and taxa above the species level differently are debated in the Discussion.

### Taxonomic meta-ontology

**Figure 3:**
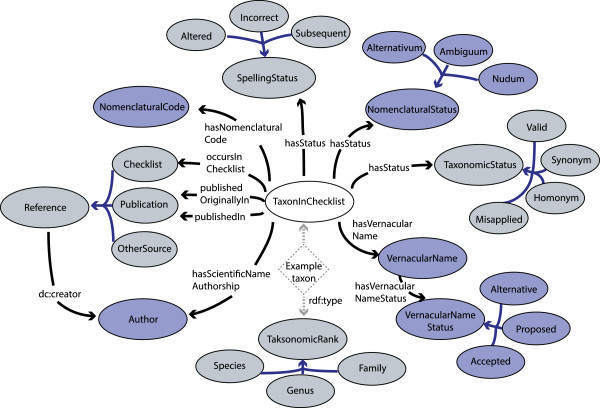
**The core classes of the taxonomic meta-ontology.** The classes are illustrated with ellipses (colours are to improve the readability of the figure). The arrows indicate relations (properties) between the classes. The subclass relations are indicated with lighter-coloured arrows and a few examples of the subclasses. To demonstrate how the TaxMeOn model is applied, an example taxon depicted using dotted lines is illustrated. The example taxon is an instance of the class *TaxonInChecklist* and of a specific taxonomic rank. The properties associated with the example taxon are marked with dotted-line arrows. The properties with literal values are not shown in the figure.

TaxMeOn is an ontology schema for biological names, and here we present the part that describes species checklists. The model is based on RDFS (RDF Schema) and some features of OWL (Web Ontology Language); it contains 12 classes with 49 subclasses (excluding 61 subclasses of the class *TaxonomicRank*) and 28 properties. The core classes and their relations are illustrated in Figure 3.

The class *TaxonInChecklist* represents both a scientific name and its concept. The relation *rdfs:label* expresses the unominal name of a taxon which is 1) the last epithet of a name combination, or 2) a name of a taxon at higher levels, e.g., a family. The taxonomic hierarchy is constructed using the relation *isPartOfHigherTaxon*.

The author references are presented in two ways:

1. The property *hasScientificNameAuthorship* expresses the author of the original publication (if the full reference of the original publication is not provided in a checklist).
2. The properties *publishedIn* and *publishedOriginallyIn* refer to the publication.

The way the taxonomic authority information is worded differs between zoology and botany. Author names are often abbreviated in diverse ways in zoology; for example, both L. and Linn. stand for Linnaeus. In botany, the abbreviations are standardised, but if a species is shifted into another genus, a new author name is catenated into the author reference (unlike in zoology). For instance, Linnaeus first described the species *Bassia scoparia* in the genus *Chenopodium* and later A.J. Scot shifted it into the genus *Bassia*. The order of multiple authors comes out in the literal, i.e., (L.) A.J. Scot.

**Figure 4:**
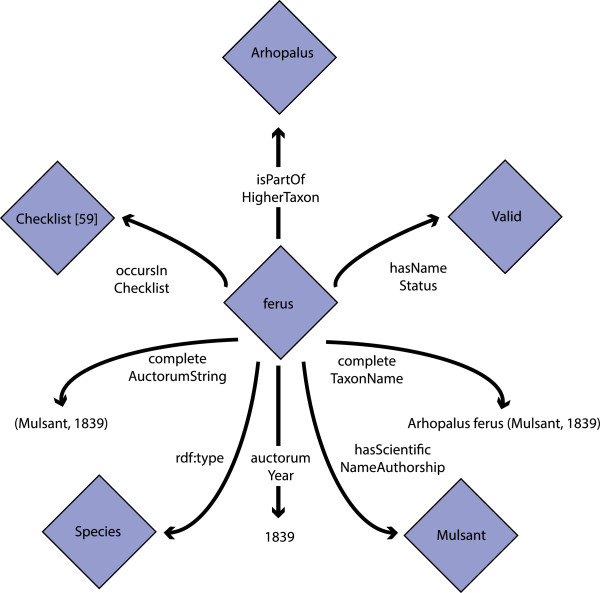
**Core taxonomic information represented according to the taxonomic meta-ontology.** *Ferus* is described by Mulsant in 1839 and it belongs to the genus *Arhopalus*.

A binominal name combination of a species with a reference to the original author (e.g., *Arhopalus ferus* (Mulsant, 1839)) is formed by traversing the RDF graph where a genus name is obtained using the *isPartOfHigherTaxon* relation and the other parts of the name from the literals. The literal *completeTaxonName* is for facilitating the usage of the model for humans, and is generated from a genus name, a species name, and an author reference. Dublin Core attributes [53] are supported (e.g., bibliographical details). Figure 4 presents an example of the species *Arhopalus ferus* which was described by Mulsant in 1839 and is a valid name. The same RDF example as Turtle [54] presentation is in Additional file 2.

In Figure 3, the relation *hasStatus* is associated with the class *TaxonInChecklist* and indicates: 1) the nomenclatural status of a name (*nomen alternativum*, *nomen correctum*, etc.), 2) the orthographic variants (altered spelling, incorrect spelling, etc.), and 3) the current opinion of a taxonomic concept (valid, synonym, etc.). Modelling the changes is further discussed in the Discussion. Other important properties and their explanations are listed in Table 2.

**Table 2:** **The core properties of the Taxonomic Meta-Ontology and their explanations**

Property	Explanation
**Citation-related properties**	
occursInChecklist	Reference to a species checklist
auctorumYear	The year of original publication
completeAuctorumString	Author name(s) expressed according
	to the established practices of taxonomy
**Name-related properties**	
hasNonvalidName	Expresses synonyms, homonyms
	and orthographic variants of a valid
	scientific name
hasVernacularName	The common name equivalents for
	the scientific names
hasNomenclaturalCode	Specifies the set of rules that are applied
	(ICN [55] or ICZN [56])
hasVernacularNameStatus	Expresses whether a common name is
	accepted or an alternative one
rdf:type	Expresses the hierarchical level in
	a classification. The ranks are obtained
	from TDWG Taxon Rank LSID Ontology [57].
	Every taxon is an instance of a specific
	taxonomic rank and the class
	*TaxonInChecklist* (Figure 3).

See other properties in the Results section, in subsection Taxonomic Meta-Ontology.

The taxonomic concepts are mapped using the relations described in Table 3. An additional relation *isAssociatedWithTaxon* is provided for linking concepts in taxonomically unresolved cases. The relation describes an undetermined connection between taxa, which is useful if deeper expertise is not available when mapping the concepts.

**Table 3:** **The relations used for mapping underlying taxonomic concepts**

Relation between taxa	Intensive	Ostensive	Notation	Properties
Congruent	Share the same characters	Share the same species	*A*=*B*	Symmetric, transitive
Part of	All characters of a taxon are included in another taxon	All species are included in another taxon	*A*⊂*B*	Non-symmetric, transitive
Overlap	At least one character is shared between taxa, but not all of them	At least one species is shared between taxa, but not all of them	*A*∩*B*≠*∅*,*A*≠*B*	Symmetric, non-transitive

The division into intensional and ostensive relations [35] is only available in TaxMeOn (not in the Taxonomic Database).

The taxa can be mapped to an external source as shown below, where the genera *Arhopalus* are mapped congruently between two checklists.

prefix cerambycids: <http://www.yso.fi/onto/cerambycids>.

prefix taxmeon: <http://www.yso.fi/onto/taxmeon/>.

cerambycids:p2090 taxmeon:congruentWithTaxonInt

urn:lsid:catalogueoflife.org:

d782a602-29c1-102b-9a4a-00304854f820:col2012acv16>.

The URI of the scientific name and its concept (*TaxonInChecklist*) is duplicated when there is a taxonomic, nomenclatural, or hierarchical change. In this way, a particular taxon can be explicitly referred to at a particular time. The old and the new URIs are connected with the relations described in Table 3. Temporal management is based on the time stamps of scientific names’ taxonomic status in dynamic checklists. In static checklists, the temporal order of the taxon instances is traced by the publication year of the checklist.

Two examples of concept mapping and taxonomic changes are presented below. Each scientific name is given a new URI in each static checklist. Different URIs for the same scientific name enable the presentation of alternative classifications and different sets of taxonomic details. The first example presents four cases presented in static checklists:

1. Two species of long-horn beetles, *pubescens* Fabricius, 1787 and *revestita* Linnaeus, 1767 belong to the genus *Leptura* Linnaeus, 1758 in the checklist that was published in 1992 [58].
2. Both species belong to the genus *Pedostrangalia* Sokolow, 1758 in the checklist published in 2011 [59].
3. The species *L. aethiops* Poda, 1761 remained in the genus *Leptura* while two other species were shifted in 2011 [59].
4. *Pedostrangalia* was a synonym for *Leptura* in 1992 [58].

The corresponding RDF representation is presented in Additional file 3.

The second example describes a fictitious dynamic checklist with three artificial taxa. The species *bus* and *cus* belonged to the genus *Aus* in 2012. Later, these two species were synonymised and *bus* remained a valid name while *cus* became its synonym. The URIs of the scientific names are duplicated in order to: 1) preserve the name combinations of the genus *Aus* (i.e., the lower-level classifications), and 2) present a change in taxonomic concepts and in status of the species *bus* and *cus*. The corresponding RDF representation is presented in Additional file 4.

The checklists are managed using the scalable generic metadata editor SAHA [60], but more complex taxonomic information of the scientific names is managed using the ontology editor Protégé [61]. The species ontologies are accessible with several user interfaces and APIs via the Finnish Ontology Library Service ONKI [42, 62]. The ONKI browser is used for searching and browsing taxa, finding currently valid names, and tracing the temporal changes in scientific names. The ONKI service also provides an autocompletion widget which can be integrated into user applications, e.g., a content management system. ONKI provides HTTP and SOAP APIs for programmatic access and a SPARQL endpoint for querying the ontologies. The checklists in ONKI are the same as in the Taxonomic Database described earlier.

The HTTP URIs were generated for the data resources in the following form: http://www.yso.fi/onto/ CHECKLIST_ID/LOCAL_ID where CHECKLIST_ID is a human-readable identifier for a checklist (or a group of checklists, if there is more than one checklist about the same group) and LOCAL_ID is a local identifier for a resource (e.g., scientific name, taxonomic status). Similarly, the URIs of the authors have namespace, with the CHECKLIST_ID replaced with the string “author”. The URIs of TaxMeOn are constructed in the same way, but the CHECKLIST_ID is replaced with the string “taxmeon”. LOCAL_ID is in the form “p[NUMBER]”, where NUMBER is a randomly generated unique identifier for the checklist data. For the authors and TaxMeOn, the LOCAL_ID is human-readable. The number of RDF triples after the data conversion (TaxMeOn) is over 1,2 million. The details are presented in Additional file 1.

TaxMeOn is applied in a broader context as one of the use cases of the European research program, the “Environmental Observation Web and its Service Applications within the Future Internet (ENVIROFI)” [63] which aims to harmonise biodiversity observation data gathered from heterogeneous sources.

## Discussion

Identifiers should not embed semantics according to the recommendations of GBIF [28, 29], a practical approach to ensure the persistence of the identifiers should the concepts change. In practise, it is helpful if URIs are intuitively understandable to some degree when reading RDF. Here, human-readable checklist identifiers are embedded in the namespace of the URIs in the data, which is justified because the namespaces are permanent. The local names of the URIs, however, do not carry meaning. The identifiers of the classes and properties in ontology models and schemas are typically human-readable, as is the case in TaxMeOn.

The HTTP URIs used in the data and in TaxMeOn act as locators for relevant metadata, that follows the best practices of Linked Data [31]. The metadata is presented as an HTML page to humans and in RDF format to machines via content negotiation.

### Comparison of the two models

The differences between the Taxonomic Database and the Taxonomic Meta-Ontology are summarised in Table 4. The Taxonomic Database is a relational database, and therefore its structure is strictly specified in a database schema. The advantage of RDF-based TaxMeOn is that it can easily be extended by adding new classes and properties. Global identifiers (URIs) are given to taxa in TaxMeOn which allows publishing them as Linked Data and linking and re-using heterogeneous data on the web. TaxMeOn can also be utilised via standard SPARQL query language and additional APIs. In contrast to the RDF model, linking other datasets to the Taxonomic Database or re-using its data is not straightforward because the data can only be accessed with a separate LSID resolver and a simple search API. The datasets of TaxMeOn can be edited with standard RDF tools, such as ontology editors, whereas the Taxonomic Database is managed with its own web interface. TaxMeOn supports more detailed taxonomic information than the Taxonomic Database, for example nomenclatural treatments. It also allows linking taxa to additional scientific publications and applying semantics to authors instead of presenting them as simple strings. Moreover, TaxMeOn provides versatile methods for managing dynamic checklists by representing temporal changes of taxonomic concepts.

**Table 4:** **A comparison of the features of the taxonomic database and the taxonomic meta-ontology**

	Taxonomic	TaxMeOn
	database	
**Technology**		
Structure easily	No	Yes
extensible		
Global linkability	No	Yes
to other contents		
Public interfaces	Simple search API,	HTTP and SOAP APIs,
	LSID resolver	Linked Data,
		SPARQL endpoint
Need of a resolver	Yes	No
Content editing	Web interface	SAHA [60], Protégé [61]
**Content**		
Granularity of	Low	High
taxonomic information		
Linking additional	No	Yes
scientific publications		
Treatment of botanical	Identical	Not identical
and zoological names		
Semantics applied to	No	Yes
author names		
Tracking temporal	Publication year	Versioning of checklists
changes	of a checklist	(static) and duplication
		of taxa (dynamic)

### Managing changes in time

In the Taxonomic Database, the goal was to create connections between the scientific names of published checklists where the timeline is evident due to the year of publication. Less emphasis was placed on dynamic lists. However, evincing temporality is achieved by tracking changes in dynamic lists, an activity that requires: 1) keeping a log of taxa removals and additions, 2) creating a new version of a checklist when taxa are removed or added, and 3) linking older LSIDs to the new ones. An original link to a genus should be kept if a species is shifted into another genus.

**Figure 5:**
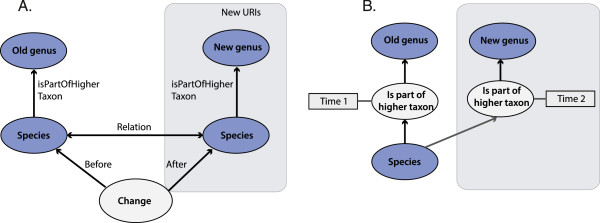
**Taxonomic changes in relation to time presented in RDF.** **(A)** The change is modelled as an instance. **(B)** The relation is modelled as an instance. The instances are depicted as lighter-coloured ellipses and literals as rectangles.

Also in TaxMeOn, the versioning of a static checklist is the solution for managing names over time given its simplicity in comparison to modelling the changes (Figure 5). Consequently, a large number of URIs are created, which is impracticable for a maintainer if special tools are not developed. Updating taxonomic changes in a dynamic checklist requires the duplication of the URIs at the species and genus level so that the situation before and after can be presented and interlinked. This step is especially necessary if there is a change in a taxonomic concept. The whole upper classification is not duplicated because that would generate a large number of URIs, and here we are more interested in names than classifications.

A machine does not understand that there was a taxonomic change if the change is not modelled. Two alternative ways of describing the changes are demonstrated in Figure 5. One approach is to present a change in a taxonomy, classification, or nomenclature by forming a class that describes the change type (Figure 5A). The situation is described before and after the change, and the two instances are connected with relevant relations (Table 3). In this way, it is possible to refer to a taxonomic concept at a particular time. An alternative approach is to represent the relations as instances (Figure 5B). The relations are ordered temporally by assigning them a time stamp. If the URIs assigned to the concepts are not duplicated, then it is not possible to refer to a taxonomic concept at a particular time. This might be practical in some cases, because a new URI is assigned only to genuinely new information (new hierarchical relations). The former alternative is included in TaxMeOn, and the latter can be used if the model is extended with an additional class that describes the relations between taxa.

### Mapping taxonomic concepts

A species checklist is an understandable way of presenting information to non-taxonomists, but unfortunately only a small proportion of species are catalogued, and they cover only limited geographical areas. Moreover, the information is often insufficient because name combinations are not necessarily listed. Cross-linking taxon names between checklists helps a user to piece together the changes in scientific names and determine the approximate number of taxonomic treatments (none vs. many). Linking higher taxa between checklists is rather artificial because the taxonomic concepts are seldom referenced. The problem is therefore how to reconcile the differing classifications of regional checklists. A pragmatic option is to compare the species included in a higher taxon. However, this approach fails to distinguish taxonomy and regionality, leading to a situation where the occurrence of a new species in a certain area changes the existing relations between the higher taxa of checklists.

The challenges of concept mapping have been discussed by many researchers [2, 35, 36, 64], and it is suggested that it should be stated whether comparisons are based on being a member of a group or on characters that unite the group [35]. In the Taxonomic Database, higher taxa are not only aligned on the basis of underlying taxonomic concepts, but the occurrence of species are also taken into account due to the lack of information about taxonomic concepts in checklists. Higher taxa of the Taxonomic Database are not mapped with the CoL’s taxa identifiers because only the part-of relation could be used (because a regional species list is always part of a worldwide list). Instead, external identifiers (CoL) are treated as additional information about the taxonomic concepts. Despite the discrepancy between taxonomy and regionality, a non-taxonomist is more likely to be interested in the species inhabiting a certain geographical area than in those found in the entire world. On the other hand, a maintainer decides how the model is applied. The taxa in both models are mapped equivalently, but TaxMeOn supports more than one way of expressing a relation between taxa (Table 3), which benefits users with differing needs and levels of expertise.

Franz and Peet [35] present how phylogenetic relationships are described using ostensive (i.e., based on being a member of a group) and intensional (i.e., based on characters) relations simultaneously, which increases the semantic precision of the relations between the concepts. In species checklists, there is no satisfactory solution to defining relations at the species level. If ostensive relations are used, there is an assumption that the species have subspecies; however, most species do not have any subspecies. Applying intensional relations assumes that the circumscriptions are known; species lists lack the information on circumscriptions. We decided to use ostensive relations as our default when mapping the concepts at the species level because the nature of the checklist can be interpreted as ostensive because they present a classification. However, intensional relations can be set if there is information about the underlying taxonomic concepts. The comparison of higher taxa (above the species level) is always based on the species (see the discussion of the Taxonomic Database above). The use of ostensive relations (Table 3) differs slightly from Franz and Peet’s [35], which is explained by the difference of the data (phylogenies vs. species checklists).

Linking the taxonomic concepts automatically is a quick way of handling datasets. Automatic mapping immediately links new content to existing without time-consuming work by experts that could be done later. A general taxon class (*TaxonGeneral*) represents a taxon at a high level of abstraction, and an instance of it is generated for all taxa. If the taxa share the same name and authorship, then they will be automatically mapped to the same instance of the class *TaxonGeneral*. The idea is to keep the machine-generated mappings separate from the manual ones. The advantage is that if the mappings are used in information retrieval, then search results can be classified according to reliability. Mistakes generated in automated work are inevitable, but most links are likely to be correct due to the non-specific nature of the class *TaxonGeneral*. Different levels of abstraction increase a model’s flexibility. For instance, the International Federation of Library Associations and Institutions’ (IFLA) [65] Functional Requirements for Bibliographic Records (FRBR) entity-relationship model [66], which is used in online library catalogues, represents the products of intellectual or artistic endeavour at four levels of abstraction.

### Challenges

Detailed information is considered more reliable and therefore more likely to be linked to other content than vague information. However, most taxonomic information in checklists is inaccurate in one way or another. Therefore the data model should support the expression of information at various levels of detail, resulting in the complexity of an ontology model. For instance, a taxonomic author citation can include a set of bibliographical details or it simply can be an abbreviation of a name. Our aim was to create a practical model that suits diverse situations, but there is a clear trade-off between practicality and complexity. Combining the scientific name and its concept into a single unit in TaxMeOn increases simplicity but decreases the granularity of information.

The biggest obstacle in using Semantic Web technologies is the lack of suitable tools. Few ontology editors are available. The most commonly used editor is Protégé, which is not practicable in this case because taxonomic classifications cannot be viewed hierarchically unless the *rdfs:subClassOf* relation is used.

In the real world, scientists who study the evolutionary relationships of organisms are often unaware of the advance of biodiversity informatics, or they simply ignore it because they evaluate the usefulness of available resources on the basis of content. Misleading or insufficient information in databases that is copied from one place to another does not encourage scientists to contribute or follow best practices. Taxonomists cannot be expected to follow what happens in biodiversity informatics because it might not be their field of interest. However, it would be very helpful if they were willing to report mistakes in content, though it would be frustrating if the corrections were not made. One can debate whether harmonising names is realistic due to the fact that scientific names are constantly changing. However, applying semantics to the content better enables the presentation of parallel views reflecting the nature of research. Databases and ontologies might not be useful for taxonomists because they rely on scientific publications, and are familiar with their own subject. Regardless, their input is fundamentally important for non-taxonomists, because the need exists for reliable taxonomic information. In general, maintaining and updating ontologies is complex compared to databases. The work is worthwhile, though, because it facilitates interoperability and the semantically enriched processing of content, and brings expert knowledge into wider use in the environmental and biological sciences.

## Conclusions

Semantic Web technologies provide a suitable way to describe species checklists because they enable the compatibility with Linked Data. This compatibility is advantageous when reusing and integrating data as well as deepening the level of biological information. Linked Data efficiently prevents the formation of silos, where distributed information is not interlinkable. The advantages of using a Semantic Web approach are presented in Table 4.

Linked Data increases the utility of data gathered from multiple sources, as their reliability is easier to evaluate. For example, the existence of multiple classifications usually indicates that a taxonomic group is complex and many opinions of it exist. Traditional databases are not compatible as such with Linked Data, and they tend to be used internally by organisations rather than shared. The structure of a relational database has to be strictly specified in advance because it cannot be easily changed later, unlike Linked Data, which is more extensible.

The next challenge is to develop a model that addresses both zoological and botanical nomenclatures that are independent of one another and separated by distinct features. We aim to develop an ontology model that covers both nomenclatures without losing the practicality. Applying Semantic Web technologies is a promising step in enhancing the linkability of biological contents and distributing environmentally important information.

## Availability of supporting data

The datasets are accessible in the Taxonomic Database, http://taxon.luomus.fi, and in the ONKI Ontology Service, http://onki.fi. The ontology schema of the TaxMeOn model is available at: http://schema.onki.fi/taxmeon/.

## Electronic supplementary material

Additional file 1: **Datasets included in the study.** (PDF 31 KB)

Additional file 2: **Core taxonomic information of a checklist expressed in RDF.** (PDF 65 KB)

Additional file 3: **Alternative classifications in static checklists expressed in RDF.** (PDF 107 KB)

Additional file 4: **A synonymisation of taxa in a dynamic checklist expressed in RDF.** (PDF 71 KB)

## Abbreviations

LSID: Life science identifier
HTTP URI: Hypertext transfer protocol uniform resource identifier
TaxMeOn: Taxonomic meta-ontology
EoL: Encyclopedia of life
CoL: Catalogue of life
GBIF: Global biodiversity information facilities
TCS: Taxonomic concept transfer schema
XML: Extensible markup language
DwC: Darwin core
TDWG: Biodiversity information standards
DwC-A: Darwin core archive
CSV: Comma-separated values
NCBI: National center for biotechnology information
URN: Uniform resource name
uBio: Universal biological indexer and organisator
RDF: Resource description framework
DNS: Domain name system
DNS SRV record: DNS service record
IANA: Assigned numbers authority
API: Application programming interface
HTML: Hypertext markup language
SPARQL: SPARQL protocol and RDF query language
NCBO BioPortal: National center for biomedical ontology BioPortal
OBO ontology language: Open biomedical ontologies ontology language
RDFS: RDF schema
OWL: Web ontology language
ICN: International code of nomenclature for algae, fungi, and plants
ICZN: International code of zoological nomenclature
SOAP: Simple object access protocol
ENVIROFI: The environmental observation web and its service applications within the future internet
IFLA: International federation of library associations and institutions
FRBR: Functional requirements for bibliographic records.
