# Supplementary material for: Making species checklists understandable to machines – a shift from relational databases to ontologies
Source: J Biomed Semantics. 2014 Sep 8;5:40. doi: 10.1186/2041-1480-5-40 (PMC4417522; doi:10.1186/2041-1480-5-40)
Supplement: Supplementary file 1 — Additional file 1: Datasets included in the study. (PDF 31 KB) [file 13326_2013_211_MOESM1_ESM.pdf]

Taxon group	Region	Publ. years	# of taxa	# of triples
Butterflies and moths (Lepidoptera)	Scandinavia, North-West Russia, Estonia	1962, 1977, 1996, 2002, 2008	313, 256, 265, 4573, 12256, 3244, 3251, 3477	470924
Thrips (Thysanoptera)	Finland	2008	219	3733
Lacewings and scorpionflies (Neuroptera and Mecoptera)	Finland	2008	113	1818
True bugs (Hemiptera)	Finland	2008	2690	44999
Flies (Diptera: Brachycera)	Finland	2008	6373	104413
Parasitic wasps (Hymenoptera: Ichneumoidae)	Finland	1995, 1999, 1999, 2000, 2003	282, 398, 919, 786, 733	49838
Bees and wasps (Hymenoptera: Apoidea)	Finland	2010	1048	17638
Mammals	World	2008	6062	227713
Birds	World	2010	12125	294547
