# Supplementary material for: Making species checklists understandable to machines – a shift from relational databases to ontologies
Source: J Biomed Semantics. 2014 Sep 8;5:40. doi: 10.1186/2041-1480-5-40 (PMC4417522; doi:10.1186/2041-1480-5-40)
Supplement: Supplementary file 2 — Additional file 2: Core taxonomic information of a checklist expressed in RDF. (PDF 65 KB) [file 13326_2013_211_MOESM2_ESM.pdf]

```
@prefix cerambycids: <http://www.yso.fi/onto/cerambycids/> .
@prefix taxmeon: <http://www.yso.fi/onto/taxmeon/> .
@prefix taxonomic-ranks: <http://www.yso.fi/onto/taxonomic-ranks/> .
@prefix rdfs: <http://www.w3.org/2000/01/rdf-schema#> .
@prefix author: <http://www.yso.fi/onto/author/> .

cerambycids:p871 # species ferus
    a taxmeon:TaxonInChecklist, taxonomic-ranks:Species ;
    rdfs:label "ferus" ;
    taxmeon:completeTaxonName "Arhopalus ferus (Mulsant, 1839)" ;
    taxmeon:isPartOfHigherTaxon cerambycids:p2090 ; # genus Arhopalus
    taxmeon:completeAuctorumString "(Mulsant, 1839)" ;
    taxmeon:hasScientificNameAuthorship author:Mulsant ;
    taxmeon:auctorumYear "1839" ;
    taxmeon:hasNameStatus cerambycids:p3555 ; # valid status
    taxmeon:occursInChecklist cerambycids:p10 . # checklist, see ref. [54]

cerambycids:p2090
    rdfs:label "Arhopalus" .

cerambycids:p3555
    a taxmeon:Valid .
```
