# Supplementary material for: Making species checklists understandable to machines – a shift from relational databases to ontologies
Source: J Biomed Semantics. 2014 Sep 8;5:40. doi: 10.1186/2041-1480-5-40 (PMC4417522; doi:10.1186/2041-1480-5-40)
Supplement: Supplementary file 3 — Additional file 3: Alternative classifications in static checklists expressed in RDF. (PDF 107 KB) [file 13326_2013_211_MOESM3_ESM.pdf]

```
@prefix : <http://www.yso.fi/onto/cerambycids/> .
@prefix taxmeon: <http://www.yso.fi/onto/taxmeon/> .
@prefix taxonomic-ranks: <http://www.yso.fi/onto/taxonomic-ranks/> .
@prefix author: <http://www.yso.fi/onto/author/> .
@prefix rdfs: <http://www.w3.org/2000/01/rdf-schema#> .
@prefix dc: <http://purl.org/dc/elements/1.1/> .
@prefix xsd: <http://www.w3.org/2001/XMLSchema#> .

:p304
    a taxmeon:Checklist ;
    dc:title "Enumeratio Coleopterorum Fennoscandiae, Daniae et
    Baltiae"^^xsd:string ;
    taxmeon:publishedInYear "1992"^^xsd:int ;
    dc:creator author:Silfverberg ;
    dc:publisher "Helsingin Hyönteisvaihtoyhdistys"^^xsd:string ;
    taxmeon:bibliographicCitation "Silferberg, H. 1992. Enumeratio
Coleopterorum
    Fennoscandiae, Daniae at Baltiae. Helsingin Hyönteisvaihtoyhdistys,
    Helsinki."^^xsd:string .

:p3396 # genus Leptura Linnaeus, 1758 in 1992
    a taxmeon:TaxonInChecklist, taxonomic-ranks:Genus ;
    rdfs:label "Leptura"^^xsd:string ;
    taxmeon:isPartOfHigherTaxon :p2835 ;
    taxmeon:hasScientificNameAuthorship author:Linnaeus ;
    taxmeon:auctorumYear "1758"^^xsd:int ;
    taxmeon:completeAuctorumString "Linnaeus, 1758"^^xsd:string ;
    taxmeon:completeTaxonName "Leptura Linnaeus, 1758"^^xsd:string ;
    taxmeon:hasNameStatus :p1252 ;
    taxmeon:hasNonvalidName :p4852 ;
    taxmeon:hasPartOst :p3399, :p2518, :p3404, :p2508 ;
    taxmeon:overlapsWithTaxonOst :p3401, :p3400, :p3402, :p3398, :p2397,
:p2514,
    :p2515, :p3051 ;
    taxmeon:refersToTaxon :p3405 ;
    taxmeon:occursInChecklist :p304 .

:p1252
    a taxmeon:Valid .

:p2523 # species L. aethiops Poda, 1761 in 1992
    a taxmeon:TaxonInChecklist, taxonomic-ranks:Species ;
    rdfs:label "aethiops"^^xsd:string ;
    taxmeon:isPartOfHigherTaxon :p3396 ;
    taxmeon:hasScientificNameAuthorship author:Poda ;
    taxmeon:auctorumYear "1761"^^xsd:int ;
    taxmeon:completeAuctorumString "Linnaeus, 1761"^^xsd:string ;
    taxmeon:completeTaxonName "Leptura aethiops Poda, 1761"^^xsd:string ;
    taxmeon:hasNameStatus :p6293 ;
    taxmeon:congruentWithTaxonOst :p2525, :p2524, :p2527, :p2526, :p2521,
:p2522 ;
```

```
    taxmeon:refersToTaxon :p2529 ;
    taxmeon:occursInChecklist :p304 .

:p6293
    a taxmeon:Valid .

:p4632 # species L. pubescens Fabricius, 1787 in 1992
    a taxmeon:TaxonInChecklist, taxonomic-ranks:Species ;
    rdfs:label "pubescens"^^xsd:string ;
    taxmeon:isPartOfHigherTaxon :p3396 ;
    taxmeon:hasScientificNameAuthorship author:Fabricius ;
    taxmeon:auctorumYear "1787"^^xsd:int ;
    taxmeon:completeAuctorumString "Fabricius, 1787"^^xsd:string ;
    taxmeon:completeTaxonName "Leptura pubescens Fabricius,
1787"^^xsd:string ;
    taxmeon:hasNameStatus :p1255 ;
    taxmeon:congruentWithTaxonOst :p4635, :p4633, :p4631, :p4630, :p4634,
:p4629 ;
    taxmeon:refersToTaxon :p1465 ;
    taxmeon:occursInChecklist :p304 .

:p1255
    a taxmeon:Valid .

:p3391 # species L. revestita Linnaeus, 1767 in 1992
    a taxmeon:TaxonInChecklist, taxonomic-ranks:Species ;
    rdfs:label "revestita"^^xsd:string ;
    taxmeon:isPartOfHigherTaxon :p3396 ;
    taxmeon:hasScientificNameAuthorship author:Linnaeus ;
    taxmeon:auctorumYear "1767"^^xsd:int ;
    taxmeon:completeAuctorumString "Linnaeus, 1767"^^xsd:string ;
    taxmeon:completeTaxonName "Leptura revestita Linnaeus,
1767"^^xsd:string ;
    taxmeon:hasNameStatus :p4853 ;
    taxmeon:congruentWithTaxonOst :p3393, :p3390, :p3392, :p3394, :p3389,
:p3387 ;
    taxmeon:refersToTaxon :p3388 ;
    taxmeon:occursInChecklist :p304 .

:p4853
    a taxmeon:Valid .

:p4852 # genus Pedostrangalia Sokolow, 1897 in 1992
    a taxmeon:TaxonInChecklist , taxonomic-ranks:Genus ;
    rdfs:label "Pedostrangalia"^^xsd:string ;
    taxmeon:hasScientificNameAuthorship author:Sokolow ;
    taxmeon:auctorumYear "1897"^^xsd:int ;
    taxmeon:completeAuctorumString "Sokolow, 1897"^^xsd:string ;
    taxmeon:completeTaxonName "Pedostrangalia Sokolow, 1897"^^xsd:string ;
    taxmeon:hasNameStatus :p1249 ;
    taxmeon:occursInChecklist :p304 .
```

```
:p1249
    a taxmeon:Synonym .

:p10
    a taxmeon:Checklist ;
    dc:title "Enumeratio renovata Coleopterorum Fennoscandiae, Daniae et
    Baltiae"^^xsd:string ;
    taxmeon:publishedInYear "2011"^^xsd:int ;
    dc:creator author:Silfverberg ;
    taxmeon:bibliographicCitation "Silfverberg, H. 2011. Enumeratio
renovata
    Coleopterorum Fennoscandiae, Daniae et Baltiae. Sahlbergia 16(2):
    1-144."^^xsd:string .

:p3399 # genus Leptura Linnaeus, 1758 in 2011
    a taxmeon:TaxonInChecklist, taxonomic-ranks:Genus ;
    rdfs:label "Leptura"^^xsd:string ;
    taxmeon:hasScientificNameAuthorship author:Linnaeus ;
    taxmeon:auctorumYear "1758"^^xsd:int ;
    taxmeon:completeAuctorumString "Linnaeus, 1758"^^xsd:string ;
    taxmeon:completeTaxonName "Leptura Linnaeus, 1758"^^xsd:string ;
    taxmeon:hasNameStatus :p4962 ;
    taxmeon:hasNonvalidName :p4959, :p4957 ;
    taxmeon:isPartOfOst :p3396, :p2514, :p2515, :p2508 ;
    taxmeon:hasPartOst :p3404, :p2508 ;
    taxmeon:overlapsWithTaxonOst :p3400, :p3402, :p2397, :p2514, :p2515,
:p3051 ;
    taxmeon:refersToTaxon :p3405 ;
    taxmeon:occursInChecklist :p10 .

:p4962
    a taxmeon:Valid .

:p2525 # species L. aethiops Poda, 1761 in 2011
    a taxmeon:TaxonInChecklist, taxonomic-ranks:Species ;
    rdfs:label "aethiops"^^xsd:string ;
    taxmeon:isPartOfHigherTaxon :p3399 ;
    taxmeon:hasScientificNameAuthorship author:Poda ;
    taxmeon:auctorumYear "1761"^^xsd:int ;
    taxmeon:completeAuctorumString "Poda, 1761"^^xsd:string ;
    taxmeon:completeTaxonName "Leptura aethiops Poda, 1761"^^xsd:string ;
    taxmeon:hasNameStatus :p4206 ;
    taxmeon:congruentWithTaxonOst :p2523, :p2524, :p2527, :p2526, :p2521,
:p2522 ;
    taxmeon:refersToTaxon :p2529 ;
    taxmeon:occursInChecklist :p10 .

:p4206
    a taxmeon:Valid .
```

```
:p2518 # genus Pedostrangalia Sokolow, 1897 in 2011
    a taxmeon:TaxonInChecklist, taxonomic-ranks:Genus ;
    rdfs:label "Pedostrangalia"^^xsd:string ;
    taxmeon:hasScientificNameAuthorship author:Sokolow ;
    taxmeon:auctorumYear "1897"^^xsd:int ;
    taxmeon:completeAuctorumString "Sokolow, 1897"^^xsd:string ;
    taxmeon:completeTaxonName "Pedostrangalia Sokolow, 1897"^^xsd:string ;
    taxmeon:hasNameStatus :p4956 ;
    taxmeon:hasNonvalidName :p4954 ;
    taxmeon:isPartOfOst :p3396, :p3704, :p3398, :p630, :p3397, :p2514,
:p2515,
    :p2511, :p2512 ;
    taxmeon:hasPartOst :p3403 ;
    taxmeon:overlapsWithTaxonOst :p3401, :p3402, :p3397 ;
    taxmeon:refersToTaxon :p4670 ;
    taxmeon:occursInChecklist :p10 .

:p4956
    a taxmeon:Valid .

:p4635 # species P. pubescens (Fabricius, 1787) in 2011
    a taxmeon:TaxonInChecklist, taxonomic-ranks:Species ;
    rdfs:label "pubescens"^^xsd:string ;
    taxmeon:isPartOfHigherTaxon :p3399 ;
    taxmeon:hasScientificNameAuthorship author:Fabricius ;
    taxmeon:auctorumYear "1787"^^xsd:int ;
    taxmeon:completeAuctorumString "(Fabricius, 1787)"^^xsd:string ;
    taxmeon:completeTaxonName "Pedostrangalia pubescens (Fabricius,
    1787)"^^xsd:string ;
    taxmeon:hasNameStatus :p4961 ;
    taxmeon:congruentWithTaxonOst :p4632, :p4633, :p4631, :p4630, :p4634,
:p4629 ;
    taxmeon:refersToTaxon :p1465 ;
    taxmeon:occursInChecklist :p10 .

:p4961
    a taxmeon:Valid .

:p3393 # species P. revestita (Linnaeus, 1767) in 2011
    a taxmeon:TaxonInChecklist, taxonomic-ranks:Species ;
    rdfs:label "reversita"^^xsd:string ;
    taxmeon:isPartOfHigherTaxon :p3399 ;
    taxmeon:hasScientificNameAuthorship author:Linnaeus ;
    taxmeon:auctorumYear "1767"^^xsd:int ;
    taxmeon:completeAuctorumString "(Linnaeus, 1767)"^^xsd:string ;
    taxmeon:completeTaxonName "Pedostrangalia reversita (Linnaeus,
    1767)"^^xsd:string ;
    taxmeon:hasNameStatus :p4281 ;
    taxmeon:congruentWithTaxonOst :p3391, :p3390, :p3392, :p3394, :p3389,
:p3387 ;
    taxmeon:refersToTaxon :p3388 ;
```

```
    taxmeon:occursInChecklist :p10 .

:p4281
    a taxmeon:Valid .
```
