# Supplementary material for: Making species checklists understandable to machines – a shift from relational databases to ontologies
Source: J Biomed Semantics. 2014 Sep 8;5:40. doi: 10.1186/2041-1480-5-40 (PMC4417522; doi:10.1186/2041-1480-5-40)
Supplement: Supplementary file 4 — Additional file 4: A synonymisation of taxa in a dynamic checklist expressed in RDF. (PDF 71 KB) [file 13326_2013_211_MOESM4_ESM.pdf]

```
@prefix : <http://example.com/> .
@prefix taxmeon: <http://www.yso.fi/onto/taxmeon/> .
@prefix taxonomic-ranks: <http://www.yso.fi/onto/taxonomic-ranks/> .
@prefix rdfs: <http://www.w3.org/2000/01/rdf-schema#> .
@prefix dc: <http://purl.org/dc/elements/1.1/> .
@prefix xsd: <http://www.w3.org/2001/XMLSchema#> .

:p1
    a taxmeon:Publication ;
    dc:date "2012-05-21"^^xsd:date .

:p2 # genus Aus in 2012
    a taxmeon:TaxonInChecklist , taxonomic-ranks:Genus ;
    rdfs:label "Aus"^^xsd:string ;
    taxmeon:completeTaxonName "Aus"^^xsd:string ;
    taxmeon:hasNameStatus :p3 ;
    taxmeon:congruentWIthTaxonInt :p9 .

:p3
    a taxmeon:Valid ;
    taxmeon:publishedIn :p1 .

:p4 # species Aus bus in 2012
    a taxmeon:TaxonInChecklist, taxonomic-ranks:Species ;
    rdfs:label "bus"^^xsd:string ;
    taxmeon:completeTaxonName "Aus bus"^^xsd:string ;
    taxmeon:isPartOfHigherTaxon :p2 ;
    taxmeon:hasNameStatus :p5 ;
    taxmeon:isPartOfOst :p11 .

:p5
    a taxmeon:Valid ;
    taxmeon:publishedIn :p1 .

:p6 # species Aus cus in 2012
    a taxmeon:TaxonInChecklist, taxonomic-ranks:Species ;
    rdfs:label "cus"^^xsd:string ;
    taxmeon:completeTaxonName "Aus cus"^^xsd:string ;
    taxmeon:isPartOfHigherTaxon :p2 ;
    taxmeon:hasNameStatus :p7 ;
    taxmeon:isPartOfOst :p11 .

:p7
    a taxmeon:Valid ;
    taxmeon:publishedIn :p1 .

:p8
    a taxmeon:Publication ;
```

```
    dc:date "2013-03-10"^^xsd:date .

:p9 #  genus Aus in 2013
    a taxmeon:TaxonInChecklist, taxonomic-ranks:Genus ;
    rdfs:label "Aus"^^xsd:string ;
    taxmeon:completeTaxonName "Aus"^^xsd:string ;
    taxmeon:hasNameStatus :p10 ;
    taxmeon:congruentWithTaxonInt :p2 .

:p10
    a taxmeon:Valid ;
    taxmeon:publishedIn :p8 .

:p11 #  species Aus bus in 2013
    a taxmeon:TaxonInChecklist, taxonomic-ranks:Species ;
    rdfs:label "bus"^^xsd:string ;
    taxmeon:completeTaxonName "Aus bus"^^xsd:string ;
    taxmeon:isPartOfHigherTaxon :p9 ;
    taxmeon:hasNameStatus :p12 ;
    taxmeon:hasNonvalidName :p13 ;
    taxmeon:hasPartOst :p4, :p6 .

:p12
    a taxmeon:Valid ;
    taxmeon:publishedIn :p8 .

:p13 #  species cus in 2013 (synonym)
    a taxmeon:TaxonInChecklist, taxonomic-ranks:Species ;
    rdfs:label "cus"^^xsd:string ;
    taxmeon:completeTaxonName "cus"^^xsd:string ;
    taxmeon:hasNameStatus :p14 .

:p14
    a taxmeon:Synonym ;
    taxmeon:publishedIn :p8 .
```
